# RETRACTION: A Meta‐Analysis of Interleukin‐6 as a Valid and Accurate Index in Diagnosing Early Neonatal Sepsis

**DOI:** 10.1111/iwj.70633

**Published:** 2025-04-23

## Abstract

**RETRACTION:** SunB. , LiangL.F. , LiJ. , YangD. , ZhaoX.B. , and ZhangK.G. , “A Meta‐Analysis of Interleukin‐6 as a Valid and Accurate Index in Diagnosing Early Neonatal Sepsis,” International Wound Journal 16, no. 2 (2019): 527–533, 10.1111/iwj.13079.30734480 PMC7948874

The above article, published online on 7 February 2019, in Wiley Online Library (http://onlinelibrary.wiley.com/), has been retracted by agreement between the journal Editor in Chief, Professor Keith Harding; and John Wiley & Sons Ltd. Following an investigation by the publisher, all parties have concluded that this article was accepted solely on the basis of a compromised peer review process. The editors have therefore decided to retract the article. The authors did not respond to our notice regarding the retraction.

**RETRACTION:** B. Sun , L.F. Liang , J. Li , D. Yang , X.B. Zhao , and K.G. Zhang , “A Meta‐Analysis of Interleukin‐6 as a Valid and Accurate Index in Diagnosing Early Neonatal Sepsis,” International Wound Journal 16, no. 2 (2019): 527–533, 10.1111/iwj.13079.30734480 PMC7948874

The above article, published online on 7 February 2019, in Wiley Online Library (http://onlinelibrary.wiley.com/), has been retracted by agreement between the journal Editor in Chief, Professor Keith Harding; and John Wiley & Sons Ltd. Following an investigation by the publisher, all parties have concluded that this article was accepted solely on the basis of a compromised peer review process. The editors have therefore decided to retract the article. The authors did not respond to our notice regarding the retraction.

